# Genetic Perturbation of the Starch Biosynthesis in Maize Endosperm Reveals Sugar-Responsive Gene Networks

**DOI:** 10.3389/fpls.2021.800326

**Published:** 2022-02-08

**Authors:** Christina Finegan, Susan K. Boehlein, Kristen A. Leach, Gabriela Madrid, L. Curtis Hannah, Karen E. Koch, William F. Tracy, Marcio F. R. Resende

**Affiliations:** ^1^Plant Molecular and Cellular Biology Program, University of Florida, Gainesville, FL, United States; ^2^Horticultural Sciences Department, University of Florida, Gainesville, FL, United States; ^3^Department of Agronomy, University of Wisconsin- Madison, Madison, WI, United States

**Keywords:** starch biosynthesis, RNA-seq, shrunken2, sugary1, sugary enhancer1, maize, endosperm, transcriptomics

## Abstract

In maize, starch mutants have facilitated characterization of key genes involved in endosperm starch biosynthesis such as *large subunit of AGPase Shrunken2* (*Sh2*) and *isoamylase type DBE Sugary1* (*Su1*). While many starch biosynthesis enzymes have been characterized, the mechanisms of certain genes (including *Sugary enhancer1*) are yet undefined, and very little is understood about the regulation of starch biosynthesis. As a model, we utilize commercially important sweet corn mutations, *sh2* and *su1*, to genetically perturb starch production in the endosperm. To characterize the transcriptomic response to starch mutations and identify potential regulators of this pathway, differential expression and coexpression network analysis was performed on near-isogenic lines (NILs) (wildtype, *sh2*, and *su1*) in six genetic backgrounds. Lines were grown in field conditions and kernels were sampled in consecutive developmental stages (blister stage at 14 days after pollination (DAP), milk stage at 21 DAP, and dent stage at 28 DAP). Kernels were dissected to separate embryo and pericarp from the endosperm tissue and 3′ RNA-seq libraries were prepared. Mutation of the *Su1* gene led to minimal changes in the endosperm transcriptome. Responses to loss of *sh2* function include increased expression of sugar (SWEET) transporters and of genes for ABA signaling. Key regulators of starch biosynthesis and grain filling were identified. Notably, this includes Class II trehalose 6-phosphate synthases, *Hexokinase1*, and *Apetala2* transcription factor-like (AP2/ERF) transcription factors. Additionally, our results provide insight into the mechanism of *Sugary enhancer1*, suggesting a potential role in regulating GA signaling via GRAS transcription factor *Scarecrow-like1*.

## Introduction

Maize (*Zea mays*) is one of the most important cereal crops in terms of economic and nutritional value and, together with rice and wheat, provides over 60% of calories consumed by people throughout the world (Fao.org). The major component of cereal seeds is the endosperm, which comprises approximately 85% of grain volume at maturity (Fao.org, 1998). The endosperm is the main site of starch synthesis and storage in the seed and serves as the source of nutrients to a germinating embryo. Starch also serves as the major calorie source for humans and livestock and is utilized in diverse industrial processes.

Starch is a storage carbohydrate primarily consisting of amylose, a linear polymer of α-(1→4)-linked glucose units, and amylopectin, a highly branched glucan polymer of α-(1→6)-linked glucose units connecting linear chains. Starch begins accumulating in the endosperm of maize kernels approximately 10 days after pollination (DAP) and continues through approximately 30 DAP, when starch production declines and kernels begin to enter the dehydration phase (reviewed in Hannah and Boehlein, 2017). Starch biosynthesis requires the coordination of multiple classes of enzymes, including adenosine 5′ diphosphate-glucose (ADP-Glc) pyrophosphorylase (AGPase), starch synthases (SSs), starch branching enzymes (SBEs), and starch debranching enzymes (DBEs) (Smith et al., 1997; Hannah and Boehlein, 2017). In maize, starch mutants have facilitated characterization of key genes involved in endosperm starch biosynthesis. Examples include *large subunit of AGPase Shrunken2* (*Sh2*) and *isoamylase type DBE Sugary1* (*Su1*). The AGPases catalyze the first committed step of starch biosynthesis and require both large and small subunits for enzymatic function. The *sh2* mutation was caused by a complex structural variation (Hu Y. et al., 2021) and produces a non-functioning endosperm AGPase. The mutation results in kernels with a severe reduction in starch content to approximately 20% of wildtype levels. Starch biosynthesis is also impacted by mutations downstream in the pathway. The *su1* allele is a mutation of Isoamylase I, which reduces starch content to approximately 50% of wildtype and shifts the ratio of amylose and amylopectin (Creech, 1968).

While many of the key enzymes involved in maize starch biosynthesis have been characterized, very little is understood about the regulation of starch biosynthesis. Some genes that affect starch biosynthesis have still not been functionally characterized. For example, the *Sugary enhancer1* (*se1*) gene is commercially relevant in sweet corn production, but its role in starch and carbohydrate metabolism is still undefined. Although its exact mechanism is unclear, the *Sugary enhancer1* mutant allele is caused by deletion of Zm00001eb115450 (Zhang X. et al., 2019). In conjunction with *su1*, the *se1* mutation further reduces the starch content and increases sugar levels, including maltose, in the kernel (Ferguson et al., 1979).

Mutations throughout the starch biosynthetic pathway provide genetic perturbations that allow us to study the transcriptional response to the changing endosperm sugar and starch levels to identify coexpression networks controlling these responses. Sugars can act as signaling molecules as well as carbohydrate sources, with regulatory control over various processes including establishment of the basal endosperm transfer layer (BETL), inflorescence branching, and activation of specific transcription factors (reviewed in Doll et al., 2017). To characterize the impact of starch mutations on the endosperm transcriptome we performed comparative RNAseq analysis on near-isogenic lines (NILs) (Wt, *sh2*, and *su1*) in six genetic backgrounds. Kernels were harvested in three consecutive developmental stages to study the responses of gene networks over time. Transcriptomes were compared to identify both conserved and background-specific differentially expressed genes (DEGs) in response to disruptions in the starch biosynthesis pathway. We show here that responses to the *sh2* mutation include increased expression of sugar (SWEET) transporters and an increase in genes involved in ABA signaling. We identify Class II trehalose 6-phosphate synthases (TPS synthetases), *Hexokinase 1*, and *Apetala2* transcription factor-like (AP2/ERF) transcription factors as potential regulators impacting the transcriptional response to *sh2*. Additionally, we propose a potential role of *ZmSE1* in regulating the hormone balance of GA and ABA to ultimately regulate starch breakdown and sugar metabolism.

## Materials and Methods

### Plant Material and Experimental Design

Eighteen sweet corn lines that included three near-isogenic lines (NILs) in six genetic backgrounds were obtained from Dr. William Tracy at the University of Wisconsin-Madison. Each inbred line, which were all originally *su1*, were backcrossed into wild type and *sh2* lines. The inbreds were selected for the corresponding genotype (wild type or *sh2*) to create near isogenic lines. Plants were grown in 2018 in Belle Glade, Florida at the University of Florida Everglades Research and Education Center in Belle Glade, FL, United States. Soil type at the research center is Histosol. Plots of 12 plants per NIL were grown in a completely randomized design with five replicates. Plants were spaced at 18 cm; rows were 76 cm apart with 0.91 m alleys between ranges. All plants were self-pollinated, and two ears were harvested from each plot at blister stage, milk stage, and dent stage (14, 21, and 28 DAP, respectively) and immediately frozen. The kernels were removed from both frozen ears, bulked per plot, and stored at −80°C for RNA extraction and quantification of starch and sugars.

### Sugar and Starch Quantification

Sugars were determined using the sucrose, D-fructose and D-glucose assay procedure from Megazyme (K-SUFRG). The assay was scaled to 96 well plate format. Briefly ears, harvested in the field, were frozen immediately in liquid nitrogen. Fifteen kernels were then taken from the equatorial region midway between the tip and base, weighed and lyophilized. The dried kernels were pulverized in a geno-grinder and 10–20 mg were transferred to each of three 1.5 mL tubes. One mL of cold water (4°C) was added to each tube and vortexed. Samples were then incubated at 80°C for 15 min. Samples were centrifuged at maximum speed for 10 min and transferred to clean 1.5 ml tubes. Appropriate dilutions adjusted sugar concentrations to within the linear range of the standard curve (0–5 μg). Typical dilutions were 1:2 for glucose and fructose, and 1:10 for sucrose. Enzymes, ATP and NADP^+^ were added according to the Megazyme assay procedure but were scaled to accommodate a 96 well format. Standard curves for glucose, fructose and sucrose were used to quantify sugars in each sample. The amount of sucrose was calculated by subtracting the amount of glucose obtained in each sample and subtracting it from the amount of sucrose plus glucose in the sample. Starch was quantified as described by Boehlein et al. (2018).

### RNA Extraction and Sequencing

For RNA sequencing, two kernels were randomly selected from each pooled-kernel sample from each of the five biological replicates and frozen in liquid nitrogen. In all the treatments, endosperm was dissected from each frozen kernel by removing the embryo, pericarp, and pedicel. Total RNA was isolated from the dissected endosperms using a CTAB based extraction as previously described (Chang et al., 1993). Samples were DNase I treated and purified using the New England Biolabs Monarch RNA Cleanup Kit (NEB). Endosperm dissection and RNA extraction were performed on each biological replicate. Quality of the RNA samples was assessed using gel electrophoresis and the three highest-quality RNA samples were then pooled in equimolar ratios for library preparation and sequencing. If there were not at least three high quality mRNA samples where 16S and 18S rRNA bands were clearly visible, the one or two high quality samples were used moving forward. This occurred only in 4 of the 52 resulting libraries were a pool of fewer than three biological replicates (Supplementary Figure S1). Libraries were prepared by the Cornell University Institute for Biotechnology using the Lexogen QuantSeq 3′ RNA-Seq Library Prep Kit FWD for Illumina according to the manufacturer’s protocol. Paired-end 75 bp reads were generated using Illumina NextSeq500.

### Reads Processing and Differential Expression Analysis

Single end reads were demultiplexed, trimmed and quality filtered using Trimmomatic v0.32 (Bolger et al., 2014). The reads were then mapped to the B73v5 reference genome (Hufford et al., 2021) using Salmon v1.1 (Patro et al., 2017). Expression was measured by quantifying transcripts per kilobase of gene per million transcripts mapped (TPM). Genes with read counts < 10 across samples were removed to reduce background. The different genetic backgrounds for the same starch mutation were treated as the replications of the alleles of interest in order to find transcriptome changes caused by the perturbation in starch biosynthesis and that are not genotype specific. Gene expression was modeled as a function of genetic background, mutation, and age of the kernel (DAP) and DESeq2 (Love et al., 2014) was used to test for differential expression with a Bonferroni-corrected *P*-value threshold of 0.05. DEGs are defined as having an absolute value log_2_ fold change > 2 unless otherwise specified.

### Identification of Coexpression Networks

Gene coexpression modules were identified using the R package Weighted Correlation Network Analysis (WGCNA) (Langfelder and Horvath, 2008). Expression data for all genes in each sample was loaded into WGCNA. The similarity matrix was raised to the power of 6 to identify coexpression modules. Once modules were constructed, module eigengene values were determined using the first principal component of the module’s expression matrix. To test for module – phenotype associations, Pearson correlations were generated for all pairwise comparisons of each module eigengene expression value and measured kernel phenotype (sucrose, glucose, fructose, starch). Bonferroni adjustments corrected for multiple comparisons.

### Gene Ontology and Pathway Enrichment Analysis

Gene Ontology (GO) terms for each gene were obtained from the B73v5eb annotation. The R package topGO v.2.38.1 was used to conduct GO enrichment analysis (Alexa and Rahnenfuhrer, 2020). GO terms with *p*-values ≤0.05 were considered over-represented. For pathway enrichment analysis, KOBAS3.0 software was used to test the statistical enrichment of DEGs in Kyoto Encyclopedia of Genes and Genomes (KEGG) pathways (Xie et al., 2011). Pathways with a Bonferroni-corrected *P*-value of ≤0.05 were considered over-represented.

## Results

### Kernel Sugar Levels Are Affected by Mutation and Genetic Background

**FIGURE 1:**
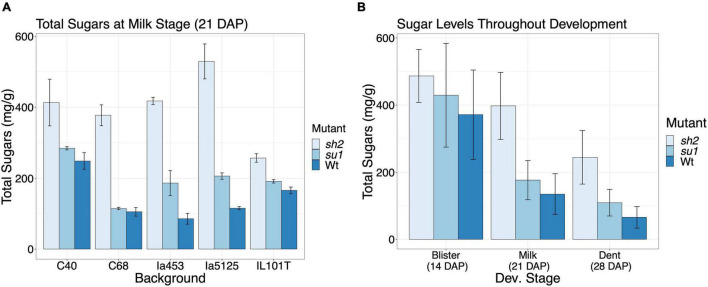
Sugar levels across NILs. **(A)** Comparison of sugar levels in different NILs at milk stage (21 DAP). Values represent the mean sum of sucrose, glucose, and fructose in percent of the dry tissue weight ± SE. **(B)** Comparison of sugar level in different mutants at different developmental stages. Values represent the mean sum of sucrose, glucose, and fructose across genotypes in percent of the dry tissue weight ± SE.

To assess the impact of starch mutations on endosperm sugar content in different maize backgrounds, we quantified sugar levels on the 18 isogenic lines (Figure 1). Milligrams of sucrose, fructose, and glucose per gram of dried tissue were quantified and summed for this analysis. At milk stage, the *sh2* mutation led to a 255.9 mg g^–1^ (*P*-value < 0.001) increase in all quantified sugars on average (Figure 1A and Supplementary Table S1). Contrastingly, the *su1* mutation resulted in an average increase in sugars of 46.12 mg g^–1^. However, this response was not consistent across genetic backgrounds. Although there is a pattern of increased sugar in *su1* kernels at each time point, when all the genotypes are jointly analyzed, there is no significant difference from the Wt sugar levels (Figure 1B and Supplementary Table S1). In most backgrounds tested, *su1* kernel sugar levels are also significantly higher than Wt by 21 DAP (*P*-value < 0.05) (Supplementary Figure S2 and Supplementary Table S1). Even taking developmental stage and mutation into account, genetic background has a significant impact on overall sugar levels (*P*-value < 0.05). Some backgrounds, most notably Ia5125, exhibit a stronger increase in sugars with the *sh2* mutation (mean sugar levels increase by 365.6 mg g^–1^) (Figure 1A and Supplementary Table S1). The sugar levels of other backgrounds are less responsive to the mutation. At 21 DAP, IL101T *sh2* kernels only have 152.3 mg g^–1^ more sugar than the wildtype kernels (Figure 1A and Supplementary Table S3). Sugar levels in the kernels decreased over time from 14 to 28 DAP (*P*-value < 0.001) (Figure 1B). By 21 DAP, *sh2* kernels have significantly higher sugar levels than *su1* or Wt kernels (*P*-value < 0.001) (Figure 1B and Supplementary Table S1).

### Generation and Analysis of RNA-Seq Data

**FIGURE 2:**
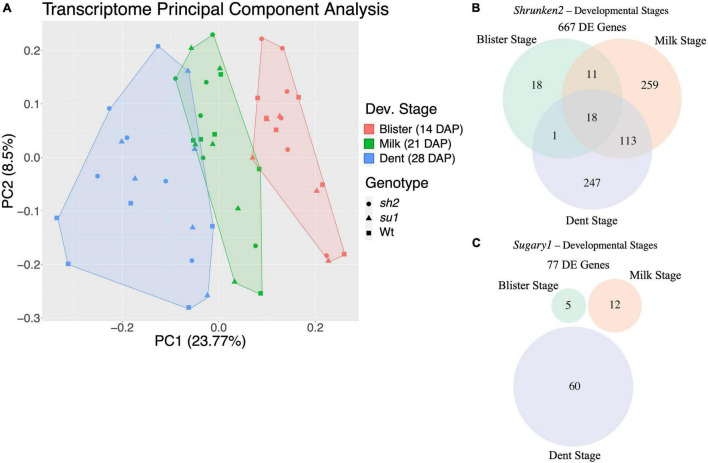
Transcriptome/DE summary. **(A)** Clusters of samples from different mutant endosperm over developmental time identified by principal component analysis of gene expression levels. Colors represent timepoints while shapes indicate mutant. **(B)** Venn diagram of DEGs in *sh2* endosperm at different timepoints. **(C)** Venn diagram of DEGs in *su1* endosperm at different timepoints.

To investigate responses to perturbations of the starch biosynthesis pathway, we compared transcriptomes from mutant and wildtype endosperm and identified DEGs. We generated 489 million high-quality single-end Illumina reads from our 52 libraries (Supplementary Figure S1). Reads were uniquely mapped to the B73v5eb transcriptome (Hufford et al., 2021) with an average mapping rate of 68.9%. Uniquely mapped reads were used to estimate normalized transcript level as transcripts per kilobase of gene per million transcripts mapped (TPM). In total, 25,972 genes were carried forward for further analysis. Principal component analysis of the TPM counts indicates that developmental stage is the most important factor in transcriptome composition (Figure 2A). Thus, all further differential expression (DE) comparisons were performed within a given developmental stage. Each genotype (*sh2*, *su1*, Wt) was represented by combining results across backgrounds. By using different genetic backgrounds as replicates, we aim to identify conserved responses to these mutations in the maize endosperm. Using an adjusted *p*-value threshold of 0.05 and a log_2_ fold change cutoff of two, 667 DEGs were identified between *sh2* and Wt endosperm and 77 DEGs were identified between *su1* and Wt endosperm in at least one stage of development (Figures 2B,C). In *sh2* endosperm, 18 genes are DE at all developmental stages examined, while in *su1* endosperm no genes are DE in all developmental stages. The 18 consistently DE genes in *sh2* endosperm include *Shrunken2*, protein kinases and chromatin regulators. The lowest number of DEGs for both comparisons (*sh2* vs. Wt and *su1* vs. Wt) is found at blister stage (Figures 2B,C). In *sh2* endosperm, the number of DEGs rapidly increases from 48 DEGs at blister stage to 401 DEGs milk stage and then slightly declines to 379 DEGs at dent stage (Figure 1B). In *su1* endosperm, there is a slight increase in DEGs between blister stage (5 DEGs) and milk stage (12 DEGs), followed by a more dramatic increase to 60 DEGs at dent stage (Figure 2C).

### Mutant vs. Wildtype Endosperm – Gene Ontology and KOBAS Enrichment

To capture broad effects of *sh2* and *su1* mutations, genes that were at least moderately differentially expressed (Bonferroni corrected *P*-value ≤ 0.05, absolute value log_2_ fold change > 1) were tested for KEGG pathway enrichment using KOBAS 3.0 (Xie et al., 2011) and GO term enrichment using the R package topGO (Alexa and Rahnenfuhrer, 2020). The *sh2* endosperm showed a downregulation of genes in the pathways for carbon metabolism and starch and sucrose metabolism. There was an upregulation of genes involved in glycolysis and gluconeogenesis, the pentose phosphate pathway, the citric acid cycle, plant hormone signal transduction, and fatty acid metabolism (Table 1 and Supplementary Table S2). The *su1* endosperm showed an upregulation of genes involved in butanoate and glutamate metabolism (Table 1 and Supplementary Table S2). GO enrichment analysis of DEGs revealed that genes up- or down- regulated in *sh2* are enriched for terms related to polysaccharide catabolism, sucrose metabolism, trehalose metabolism, 1,3-beta-D-glucan synthesis, glycolysis, and lipid biosynthesis (Supplementary Table S3). Genes up- and down-regulated in *su1* are enriched for terms related to peptidase activity, carboxylyase activity, carboxylic acid metabolic process, metal ion transport, and malate dehydrogenase activity are significantly enriched (Supplementary Table S3).

**TABLE 1:** KOBAS pathway enrichment results.

Mutant	Expr. change	KEGG pathway name	Pathway ID
*sh2*	Down	Starch and sucrose metabolism	zma00500
		Metabolic pathways	zma01100
		Photosynthesis	zma00195
		Plant hormone signal transduction	zma04075
		2-Oxocarboxylic acid metabolism	zma01210
		Carbon metabolism	zma01200
	Up	Metabolic pathways	zma01100
		Biosynthesis of secondary metabolites	zma01110
		Carbon metabolism	zma01200
		Glycolysis/gluconeogenesis	zma00010
		Biosynthesis of amino acids	zma01230
		Pyruvate metabolism	zma00620
		Amino sugar and nucleotide sugar metabolism	zma00520
		Fatty acid metabolism	zma01212
		Purine metabolism	zma00230
		Alpha-linolenic acid metabolism	zma00592
		Starch and sucrose metabolism	zma00500
		Fructose and mannose metabolism	zma00051
		Fatty acid biosynthesis	zma00061
		Glycerophospholipid metabolism	zma00564
		Fatty acid degradation	zma00071
		Butanoate metabolism	zma00650
		Glyoxylate and dicarboxylate metabolism	zma00630
		Phenylpropanoid biosynthesis	zma00940
		Cysteine and methionine metabolism	zma00270
		Carbon fixation in photosynthetic organisms	zma00710
		Selenocompound metabolism	zma00450
		Flavonoid biosynthesis	zma00941
		Terpenoid backbone biosynthesis	zma00900
		Fatty acid elongation	zma00062
		Biosynthesis of secondary metabolites - unclassified	zma00999
		Diterpenoid biosynthesis	zma00904
		Synthesis and degradation of ketone bodies	zma00072
		Plant hormone signal transduction	zma04075
		Plant–pathogen interaction	zma04626
		Benzoxazinoid biosynthesis	zma00402
		Tryptophan metabolism	zma00380
		Citrate cycle (TCA cycle)	zma00020
		Pentose phosphate pathway	zma00030
		Ubiquinone and other terpenoid-quinone biosynthesis	zma00130
*su1*	Down	Butanoate metabolism	zma00650
	Up	NA	NA

### Differential Expression in *su1* Kernels

A total of 77 genes were significantly DE between *su1* and wildtype endosperm, 18 of which overlap with DEGs in *sh2* endosperm when compared to wildtype. The transcriptomic profiles between wildtype and *su1* kernels were most different at dent stage (28 DAP), near the completion of starch synthesis (Supplementary Table S4). At this timepoint, out of the list of DEG, an alcohol dehydrogenase Zm00001eb317510 is upregulated with a log_2_ fold-change of 2.9. Furthermore, transcription factors exhibit differential regulation in *su1* endosperm. AP2/ERF transcription factor *ZmEREB4* is upregulated with a log_2_ fold-change of 3.1, while transcription factors *ZmHB37* and *ZmC3H39* are downregulated (log_2_ fold-change of −2.2 and −2.5, respectively). An F-box containing protein (Zm00001eb067000) is significantly downregulated in *su1* endosperm with a log2 fold-change of −7.1. Additionally, a number of auxin related genes including *ZmIAA24* are downregulated in *su1* endosperm (log_2_ fold-change = −2.8). Interestingly, the aforementioned alcohol dehydrogenase and *ZmEREB4* are also upregulated in *sh2* endosperm (Supplementary Tables S4, S5).

### Impacts of the *sh2* Mutation on Carbohydrate and Sugar Metabolism Gene Expression

**FIGURE 3:**
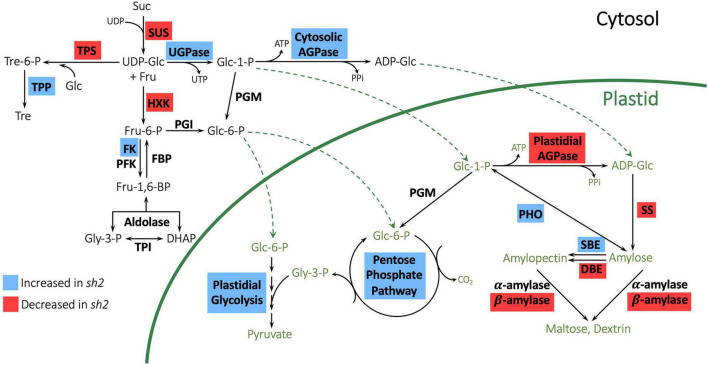
Expression changes of genes in the starch biosynthetic pathway in maize endosperm. Enzymes with gene expression changes in *sh2* endosperm are indicated with colored backgrounds. Blue boxes indicate increased expression in sh2 endosperm, while red boxes indicate decreased expression in *sh2* endosperm. Suc, sucrose; Fru, fructose, Glc, glucose; Tre, trehalose; Glc-1-P, glucose 1-phosphate; Glc-6-P, glucose 6-phosphate, Fru-6-P, fructose 6-phosphate; Fru-1,6-BP, fructose 1,6-bisphosphate; Tre-6-P, trehalose 6-phosphate; Gly-3-P, glycerol 3-phosphate; DHAP, dihydroxyacetone phosphate; PPi, pyrophosphate; ADP-Glc, ADP glucose; UDP-Glc, UDP glucose; SUS, sucrose synthase; UGPase, UDP-glucose pyrophosphorylase; AGPase, ADP-glucose pyrophosphorylase; HXK, hexokinase; FK, fructokinase; PFK, phosphofructokinase; FBP, fructose 1,6 bisphosphatase; TPI, triose phosphate isomerase; PGM, phosphoglucomutase; PGI, phosphoglucoisomerase; PHO, starch phosphorylase; SS, starch synthase; SBE, starch branching enzyme; DBE, debranching enzyme; TPS, trehalose-6-phosphate synthase; TPP, trehalose-6-phosphate phosphatase.

**FIGURE 4:**
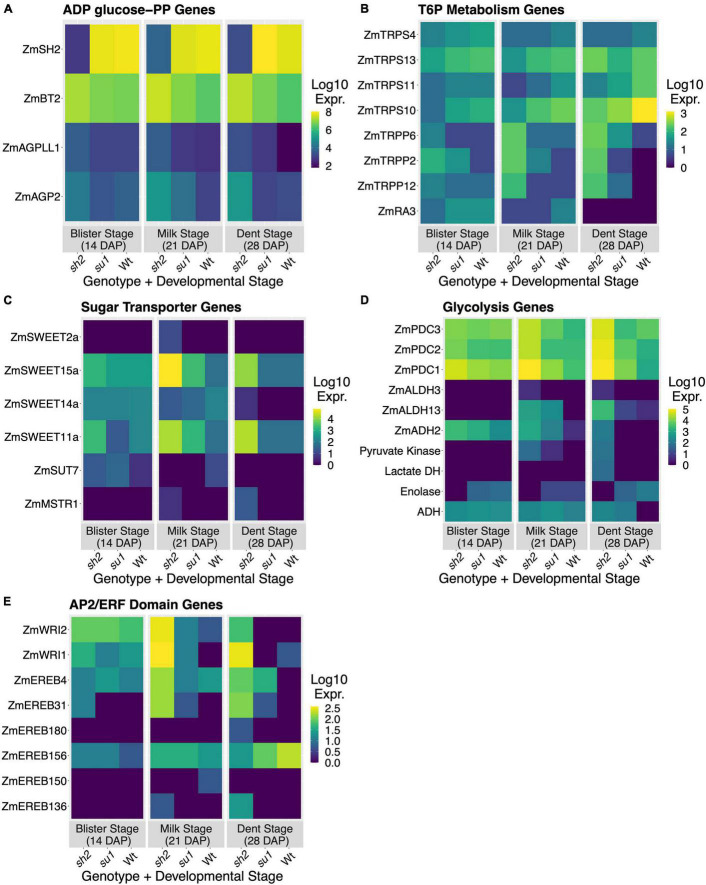
Differential expression in maize endosperm. Relative expression levels of DEGs in each mutant/timepoint combination. Values shown represent the log_10_ of the TPM summed across transcripts and averaged across backgrounds. **(A)** Relative expression of AGPase-encoding genes. **(B)** Relative expression of trehalose-6-phosphate metabolizing enzymes. **(C)** Relative expression of sugar-transporting genes. **(D)** Relative expression of genes encoding glycolytic enzymes. **(E)** Relative expression of AP2/ERF domain transcription factors.

Many genes involved in sugar and carbohydrate metabolism were differentially expressed in response to the *sh2* mutation (Table 1 and Supplementary Tables S2, S3, S5). To further characterize changes to the pathway, we investigated expression changes of genes in the KEGG starch and sucrose metabolism pathway (Figure 3). The strongest downregulation in response to the *sh2* mutation is that of the *Shrunken2* gene itself with an average log_2_ fold change of −7.15. Other AGPases including *ZmAGP2* (log_2_ fold change = 2.06) and Brittle2 (log_2_ fold change = 1.34) are upregulated in the *sh2* mutant (Figures 3, 4A). A UDP glucose pyrophosphorylase (UDPGP) is also upregulated (log_2_ fold change = 2.3) in *sh2* endosperm (Figure 3 and Supplementary Table S5). Genes involved in starch synthesis and starch degradation, including *Granule-bound starch synthase2* (*ZmGBSS2*), *Pullulanase* (*ZmZPU1*), and multiple β-amylases show significantly decreased expression in *sh2* endosperm. Starch phosphorylase *ZmPHO1* is slightly upregulated in *sh2* endosperm (log_2_ fold change = 1.1) (Figure 3 and Supplementary Table S5). Notably, genes involved in trehalose metabolism show differential expression in response to the *sh2* mutation. *Trehalose-6-phosphate (T6P) synthetase 11* (*ZmTRPS11*) is downregulated in *sh2* endosperm while *T6P phosphatase2* (*ZmTRPP2*) is upregulated (Figures 3, 4B). In addition to sugar and starch metabolism genes, sugar transporters are differentially expressed in *sh2* endosperm (Figure 4C). Sugar equilibrators including *ZmSWEET15a*, *ZmSWEET11a*, and *ZmSWEET2a* are significantly upregulated in *sh2* endosperm. Contrastingly, sucrose transporter *ZmSUT7* is significantly downregulated in *sh2* endosperm (Figure 4C).

### The *sh2* Mutation Increases Glycolysis and Pentose-Phosphate Pathway Gene Expression

As presented above, the large increase in sucrose and hexose content in *sh2* endosperm results in a significant enrichment of DEGs in the glycolysis and gluconeogenesis pathway and in the pentose phosphate pathway (Table 1 and Supplementary Table S2). Most DEGs in the glycolysis and gluconeogenesis pathway are upregulated. DEGs include alcohol dehydrogenase *ZmADH2* (log_2_ fold change = 2.1), aldehyde dehydrogenases *ZmALDH3* and *ZmALDH13* (log_2_ fold change = 2.5, 2.8, respectively), a pyruvate kinase (Zm00001eb433740; log_2_ fold change = 2.42), a pyruvate-phosphate dikinase (Zm00001eb278140; log_2_ fold change = 2.15), and a moderate upregulation (average log_2_ fold change = 1.2) of the three annotated maize pyruvate decarboxylases (*ZmPDC1*, *ZmPDC2*, *ZmPDC3*) (Figure 4D and Supplementary Table S5). Interestingly an enolase (Zm00001eb098760) is the only part of the glycolytic pathway significantly downregulated (log_2_ fold change −2.1) (Figure 4D). The pentose phosphate pathway is also significantly enriched with DEGs. Twelve genes in the pentose phosphate pathway are DE in *sh2* endosperm. Eleven of these genes are moderately but significantly upregulated (average log_2_ fold change = 1.34) (Supplementary Table S5). These include transketolases, a transaldolase, pyruvate dehydrogenase, phosphofructokinases, and phosphogluconate dehydrogenase *ZmPGD4*. The exception to this trend is a ribose-5 phosphate isomerase, which is downregulated in *sh2* endosperm (log_2_ fold change = −1.4) (Supplementary Table S5). Taken together, this indicates that the upregulation of genes in the pentose phosphate and glycolytic pathways are important components of the response to *sh2* and/or the increased sucrose levels in the endosperm.

### Differential Expression of *Apetala2* Transcription Factor-Like Transcription Factors in *sh2* Endosperm

Several genes involved hormone response are DE in *sh2* endosperm. Notably, eight *Apetala2* transcription factor-like (AP2/ERF) domain transcription factors are differentially expressed in the *sh2* endosperm (Figure 4E). Six AP2/ERF TFs are upregulated (*ZmWri1*, *ZmWri2*, *ZmEREB4*, *ZmEREB31*, *ZmEREB136*, *ZmEREB180*) in the *sh2* endosperm while two (*ZmEREB150*, *ZmDBP4*) are downregulated (Figure 4E). An additional AP2/ERF TF, *ZmEREB156*, exhibited moderate downregulation in sh2 endosperm with a log_2_ fold-change of −1.8 at 28 DAP (Figure 4E). Previously identified target genes of ZmWri1 (Pouvreau et al., 2011) show moderate (1 < log_2_ fold-change < 2) but significant upregulation in *sh2* endosperm. These targets include *ZmPYK3*, pyruvate dehydrogenases (Zm00001eb050410, Zm00001eb031900, Zm00001eb138800), acyl carrier proteins (Zm00001eb331920, Zm00001eb102360), *ZmTHL1*, and *ZmGLPDH4* (Supplementary Table S5).

### Zein-Encoding Genes and Nitrogen Related Genes Have Altered Expression in *sh2* Endosperm

Zeins compose the majority of storage proteins in the maize endosperm and are encoded by a large gene family (reviewed in Li and Song, 2020). To further explore the dynamics of protein biosynthesis in *sh2* endosperm, we evaluated the expression of zein-encoding and other protein-related gene families. Overall, expression changes in zeins and amino acid transporters indicate differential regulation of grain filling in *sh2* endosperm. Twelve zeins, including *ZmFloury4*, are downregulated in *sh2* endosperm (log_2_ fold change between −2.1 and −5.6) (Supplementary Table S5). Most (8 of 12) of the differentially regulated zeins are annotated 19 kD or 22 kD alpha zeins, except for Zm00001eb313790, a 50 kD gamma zein. An endosperm-expressed nitrate transporter1/peptide transporter family (NPF) protein (Zm00001eb112670) and a transmembrane amino acid transporter (Zm00001eb257490) are also downregulated in *sh2* endosperm (log_2_ fold change −2.2, −2.6, respectively). However, a homolog of *AtAAP1* (Zm00001eb384590) and nitrate-reductase *ZmNNR2* are upregulated (log_2_ fold change 2.6, 2.3, respectively) (Supplementary Table S5). The GABA shunt also plays a role in maintaining the carbon:nitrogen balance in the cells (Fait et al., 2008; Batushansky et al., 2014). Two glutamate decarboxylases (Zm00001eb399220, Zm00001eb081550), which catalyze first step in the GABA shunt, are upregulated in *sh2* endosperm (log_2_ fold change 1.6, 2.1, respectively) (Supplementary Table S5).

### Genetic Background Affects Sugar Accumulation in *sh2* Kernels

To gain insight into the background effect of sugar accumulation in the *sh2* endosperm, DEGs were compared across genetic backgrounds. Backgrounds were classified as “high-sugar-accumulating” or “low-sugar accumulating” based on whether the increase in sugars of *sh2* kernels was significantly above or below the average change (+255.9 mg g^–1^) (Figure 1A, Supplementary Figure S2, and Supplementary Table S1). Most notably, core histone *ZmHIS2B5* is downregulated in the low-sugar-accumulating backgrounds IL101T and C40 (log_2_ fold change = −3.7, −6.2, respectively) and either slightly upregulated (Ia453, log_2_ fold change = 1.6) or not significantly DE in high-sugar-accumulating genotypes (Supplementary Table S6). A helix-loop-helix transcription factor (Zm00001eb101410; *ZmBHLH157*) is specifically upregulated in low-sugar-accumulating backgrounds (Table 2 and Supplementary Table S6). Other expression changes are specific to high-sugar-accumulating backgrounds, including the downregulation of a protein of unknown function (Zm00001eb250800) and a protein tyrosine kinase (Zm00001eb158770; *ZmMKKK55*) and the upregulation of a glutathione S-transferase (Zm00001eb397280) (Table 2 and Supplementary Table S6).

**TABLE 2:** Differential expression of putative modifiers.

B73v5eb	Gene	Log_2_ fold-change in *sh2* endosperm					Encoded protein domain
		High-sugar-accumulating background			Low-sugar-accumulating background		
		C68	Ia453	Ia5125	C40	IL101T	
Zm00001eb188570	*ZmHIS2B5*	NA	1.64	NA	–6.22	–3.74	Core histone H2A/H2B/H3/H4
Zm00001eb101410	*ZmBHLH157*	NA	NA	NA	1.03	1.04	Helix-loop-helix DNA-binding domain
Zm00001eb250800	NA	–2.87	–2.66	–3.06	NA	NA	Unknown function
Zm00001eb158770	*ZmMKKK55*	–2.16	–1.64	–2.22	NA	NA	Protein tyrosine kinase
Zm00001eb397280	NA	3.50	4.84	1.46	NA	NA	Glutathione *S*-transferase; C-term domain

### Coexpression Networks in the Maize Endosperm Identified Nine Modules With an Overrepresentation of Carbohydrate Metabolism Genes

**FIGURE 5:**
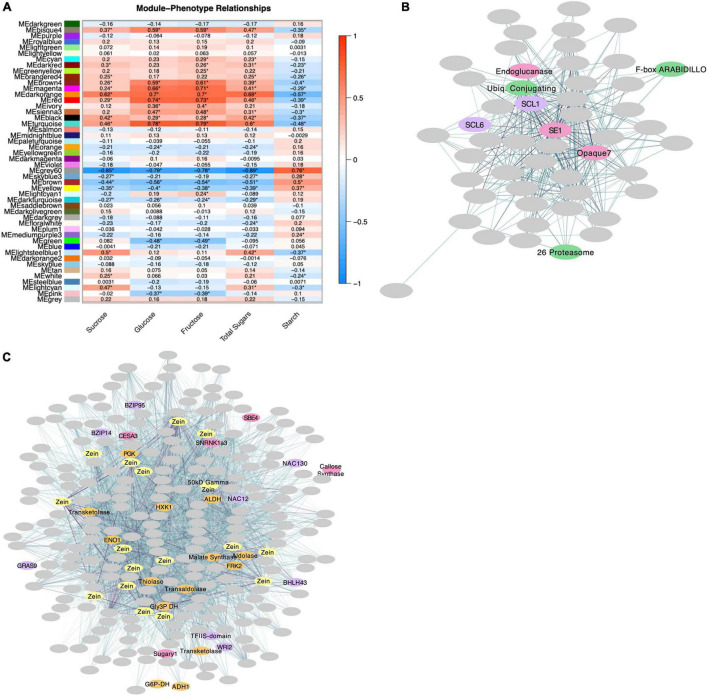
WGCNA results. **(A)** Pairwise correlations between module eigengenes and phenotypic values. Significance (*P*-value < 0.05) is indicated with an asterisk. **(B)** Visual representation of Module Lightsteelblue1 using the yFiles organic layout format. SCF-complex genes are indicated in green, genes that impact grain filling and starch metabolism are indicated in pink, and transcription factors in purple. Connection weight is indicated by line color, where darker lines represent higher-weighted connections. **(C)** Module Grey60 represented by Cytoscape Network Analyzer using the yFiles organic layout format. Genes encoding zein proteins are indicated in yellow, genes involved in carbohydrate metabolism are indicated in pink, transcription factors in purple and glycolytic genes in orange. Connection weight is indicated by line color, where darker lines represent higher-weighted connections.

Expressed genes were classified into 48 coexpression modules using the WGCNA R Software package (Langfelder and Horvath, 2008) and modules were assigned a color for naming and visualization purposes (as seen in Figure 5A). Module sizes ranged from 50 to 2643 genes with an average size of 541. Analysis for GO Term enrichment using topGO (Alexa and Rahnenfuhrer, 2020) revealed nine modules with an overrepresentation of carbohydrate metabolism genes. These modules were carried forward for further analysis using Cytoscape’s Network Analyzer (Assenov et al., 2008). Module eigengenes were calculated, and pairwise correlations with sugar- and starch-level phenotypes were generated (Figure 5A). Module Black, which contains an overrepresentation of genes related to trehalose metabolism (GO:0005992), pyruvate kinase activity (GO:0004743), G3P metabolism (GO:0004367; GO:0046168), oligosaccharide biosynthesis (GO:0009312), and cellulose metabolism (GO:0016760; GO:0030244) (Supplementary Table S7), has its eigengene positively associated with sugar levels (sucrose = 0.42, glucose = 0.29, fructose = 0.28) and negatively associated with starch levels (−0.37) (Figure 5A). In this module, *ZmTRPS10* has the highest betweenness centrality, suggesting a central role in regulating this group of genes (Supplementary Table S8). Other modules including Lightsteelblue1 and Grey60 show strong correlations with sugar and starch levels as well. Lightsteelblue1’s eigengene is associated with sucrose (0.5), starch (−0.37), and adjusted sugars (0.28) (Figure 5A). Lightsteelblue1 is a module consisting of 75 genes (Supplementary Table S9). *Sugary enhancer1* (*ZmSE1*, Zm00001eb115450) is a key regulator of this network with the highest closeness centrality and betweenness centrality values. The hub gene, or gene with the highest number of connections to other genes in the module, is the GRAS transcription factor Scarecrow-like 1 (*ZmSCL1*, Zm00001eb344400). Module Lightsteelblue1 appears to be involved in the regulation of genes at the protein level with enriched GO Terms related to protein phosphatase regulatory activity and an overrepresentation of genes in the KEGG RNA transport pathway (specifically translation initiation) (Supplementary Table S3). Lightsteelblue1 contains a number of genes related to ubiquitin-mediated protein degradation including 26 proteasome component Zm00001eb232220, ubiquitin conjugating enzyme Zm00001eb295710, and F-box protein *ZmARABIDILLO1* (Zm00001eb023660) (Figure 5B). Module Grey60 has the strongest association with sugar levels – its eigengene is significantly associated with sucrose (−0.85), glucose (−0.79), fructose (−0.78), starch (0.76), adjusted sugars (−0.37) and adjusted starch (0.29) (Figure 5A). Module Grey60 consists of 265 genes and is enriched with genes related to glycolysis (GO:0006096) and the non-oxidative branch of the pentose-phosphate pathway (GO:0009052) (Supplementary Tables S7, S8). The gene with highest betweenness centrality in this module is *ZmHXK1*. The hub gene, or most connected gene, of Module Grey60 is a 50 kD Gamma zein Zm00001eb313790. There are 15 additional zein genes in this module (Figure 5C and Supplementary Table S8).

### Differential Expression in *se1* Kernels

To further investigate the role of Se1 in transcriptional regulation, we compared RNAseq profiles from two replicates of wildtype and *se1* kernels sampled at 11, 15, and 19 DAP (Zhang X. et al., 2019). We downloaded RNAseq data from via the National Center for Biotechnology Information Sequence Read Archive BioProject PRHNA287265 (Zhang X. et al., 2019). Reads were trimmed and uniquely mapped to the B73v5 transcriptome (Hufford et al., 2021) with an average mapping rate of 83.8%. Low-count transcripts (transcripts with <10 counts total) were removed, leaving 25964 genes for further analysis. The R package DESeq2 was used to identify 116 total DEGs (log_2_ fold change > 2, Bonferroni-corrected *P*-value < 0.05) between *se1* and Wt kernels over the three timepoints. Six DEGs were identified between Wt and *se1* kernels at 11 DAP, 12 DEGs at 15 DAP, and 112 DEGs at 19 DAP (Supplementary Table S9). Certain regulatory genes are oppositely DE (compared to Wt) in *se1* kernels and *sh2* endosperm. A homolog of *AtSNF4* (Zm00001eb139880) is upregulated in *sh2* and downregulated in *se1* kernels, while the GRAS transcription factor *ZmSCL1* is upregulated in *se1* kernels and downregulated in *sh2* endosperm. In addition to *ZmSCL1*, other gibberellin-related genes are DE in *se1* kernels. A gibberellin degradation enzyme, GA4 2β-hydroxylase (Zm00001eb412670), is the most upregulated gene in *se1* kernels (log_2_ fold change = 7.07). *B-glucosidase 6*, a gene involved in gibberellin-ester hydrolysis, is also upregulated in *se1* kernels (log_2_ fold change = 2.7). Additionally, starch degradation genes known to be activated by GA (MacLeod and Millar, 1962) are upregulated in *se1* kernels (Endoglucanase, Zm00001eb015719, log_2_ fold change = 1.67) (Supplementary Table S9). It is also notable that genes related to galactose and raffinose metabolism are DE in *se1* kernels. *ZmRAFS3* is downregulated (log_2_ fold change = −8.9) at the 19 DAP timepoint in *se1* kernels while another raffinose synthase (Zm00001eb005800) is upregulated at this timepoint (log_2_ fold change = 2.7). Additionally, two β-galactosidases (Zm00001eb152540, Zm00001eb383110) are upregulated in *se1* kernels at 19 DAP (log_2_ fold change = 5.3, 4.5, respectively) (Supplementary Table S9).

## Discussion

### Mutation and Timepoint Impact the Endosperm Transcriptome

*Shrunken2* encodes the large subunit of the endosperm AGPase, responsible for synthesis of ADP-glucose used for starch production in the kernel. The mutant, *sh2*, is a complete knock out of this gene, and as such creates a drastic kernel phenotype where kernels have ∼25% wildtype starch content and a shriveled (shrunken) pericarp (Hannah, 1997). *Sugary1* encodes maize isoamylase I. Mutations in *su1* have a less severe impact on starch production and grain filling in the kernels. Kernels have approximately 50% of their wildtype starch content. The total number of DEGs between *sh2* and wildtype endosperm is much larger than DEGs between *su1* and wildtype (667 DEGs vs. 77 DEGs) (Figures 2B,C and Supplementary Tables S4, S5). This result is consistent with expectations due to the severity of the impact *sh2* has on the kernel development and endosperm composition. Starch biosynthesis and sugar accumulation begins at ∼10 DAP and continues until ∼30 DAP. We isolated mRNA from wildtype and mutant (*sh2* and *su1*) endosperm at multiple developmental stages spanning starch biosynthesis and sugar accumulation (blister stage at 14 DAP, milk stage at 21 DAP, and dent stage at 28 DAP). Developmental timepoint had a strong impact on the overall transcriptome profile (Figure 2A). As such, we analyzed each developmental stage separately to identify DEGs between mutant and wildtype kernels. Interestingly, only 18 genes were differentially regulated in *sh2* endosperm at all time points. These genes include protein kinases and chromatin remodeling proteins, suggesting that part of the changes in *sh2* endosperm may be post-translationally and/or epigenetically regulated (Figure 2B). Between *sh2* and wildtype endosperm, the number of DEGs increased from 48 at 14 DAP to 401 at 21 DAP (Figure 2B and Supplementary Table S5). In wildtype kernels, activity of the *Shrunken2* enzyme activity peaks at approximately 22 DAP (Tsai et al., 1970). During this developmental stage, we also see the largest difference in sugar content between *sh2* and wildtype kernels (Figure 1B). The differences in the transcriptomes are also more pronounced at this timepoint. Contrastingly, DEGs between *su1* and wildtype endosperm peaked later at 28 DAP (Figure 2C and Supplementary Table S4).

### Genetic Background Effects Impact Phenotypic Response to Mutations

Mutations in the starch biosynthetic pathway have variable effects depending on the genetic background (Shuler et al., 2017). To assess the degree of difference between our isogenic lines, sugar levels were quantified in kernels at three key stages of development. The *sh2* mutation resulted in a strong increase in sugars compared to both wildtype and *su1* kernels in all genetic backgrounds at 21 and 28 DAP (Figures 1A,B, Supplementary Figure S2, and Supplementary Table S1). The *su1* mutation, however, resulted in a more subtle sugar increase. In fact, when all genotypes are taken together, the *su1* mutation does not significantly increase sugar levels (Figure 1B and Supplementary Table S1). This is due to the variability in response to the mutation between genetic backgrounds (Supplementary Figure S2). The large effect of genetic background potentially explains the lack of significant DEGs in *su1* endosperm when results are combined across backgrounds. Genotypic background variability is observed in the *sh2* response as well. In some genotypes, the increase in sugars is less dramatic and not significant until later in development (Supplementary Figure S2 and Supplementary Table S1). In addition to the *sh2* and *su1* mutations, many different regulators or modifiers of the starch biosynthetic pathway have been identified, including the transcription factor, *ZmNAC130* (Zhang Z. et al., 2019). Variability of backgrounds in their sugar accumulation (Supplementary Figure S2 and Supplementary Table S1) allowed us to further pursue such putative modifiers of endosperm composition in *sh2* kernels. To identify putative regulators and modifiers, we searched for genes whose expression changes correlate with sugar accumulation in response to *sh2* (Supplementary Table S6). Notably, histone *ZmHIS2B5* and MAPKKK protein *ZmMKKK55* expression profiles correlate to low- and high-sugar-accumulating backgrounds, respectively, indicating a potential regulatory role modulating the impact of *sh2* (Table 2 and Supplementary Table S6).

### The *sh2* Mutation Alters Expression Profiles of Genes in the Starch Metabolism Pathway

Pathway and GO term enrichment analysis reveal a significant overrepresentation of starch biosynthesis and degradation genes that are differentially regulated in *sh2* kernels. As *sh2* disrupts the first committed step of starch biosynthesis, many of the DEGs involved in starch synthesis are downregulated (Table 1 and Supplementary Table S2). Starch synthases and starch degradation genes, including amylases, are also downregulated (Figure 3), likely due to the increase in sugars in the endosperm enhancing sink activity (Bihmidine et al., 2013; Guan and Koch, 2015). *Shrunken2* transcripts are observed at much lower levels with an average log2 fold change of −7.15 (Figure 4A). Other AGPases, including *Brittle2* (*ZmBT2*) are upregulated in the *sh2* endosperm (Figure 4A), which corroborates previous findings (Giroux et al., 1994). In addition to other AGPases, a UDP-glucose pyrophosphorylase (UDPGP) gene is upregulated in *sh2* endosperm (Supplementary Table S5), possibly due to an increased concentration of glucose 1-phosphate. Interestingly, certain starch synthases have demonstrated activity using UDP-Glucose as a substrate, although at much lower levels than ADP-Glucose (Macdonald and Preiss, 1983). *Starch phosphorylase* 1 is upregulated in *sh2* endosperm (Figure 3 and Supplementary Table S5). *Starch phosphorylase1* (*ZmPHO1*) is the plastidial starch phosphorylase and is typically active in the endosperm amyloplasts (Yu et al., 2001; MaizeGDB.org, 2021). The function of starch phosphorylase in maize kernels is unclear, as evidence has suggested a role in both degradative and synthetic processes (Subasinghe, 2013). Under specific conditions, disruptions in starch phosphorylase activity have resulted in reductions in starch accumulation (Satoh et al., 2008). ADP-glucose typically inhibits starch phosphorylase activity in the synthetic direction during later developmental stages (Hwang et al., 2010). Upregulation of *ZmPHO1* in *sh2* endosperm in later stages of kernel development further supports the hypothesis that in the absence of AGPase function, *ZmPHO1* could be responsible for the remaining 25% starch production in the endosperm (Boehlein et al., 2018).

### Sugar-Responsive Genes for Sink Strength

Among the genes for source-sink adjustment affected here are those aiding sugar transport, with upregulation of mRNAs for sugar equilibration in sinks versus downregulation of those related to phloem loading in sources (SWEETs and SUTs, respectively). Members of the SWEET (Sugars Will Eventually be Exported Transporter) protein family transport sucrose and/or hexoses in the direction of the concentration gradient (Chen et al., 2010). Members of the SUT (sucrose transporter) protein family transport sucrose against the concentration gradient and are often involved in phloem loading (reviewed in Braun and Slewinski, 2009). The SWEET genes upregulated in *sh2* endosperm (Figure 4C) include *SWEET2*, a member of the glucose-equilibrating Clade I, and two members of the sucrose-equilibrating Clade III (*SWEET11a* and *SWEET15a*; Eom et al., 2015). In contrast, the *SUT7* downregulated in *sh2* endosperm (Figure 4C) has a close homolog, *SUT1*, with a demonstrated role in phloem loading (reviewed in Julius et al., 2017).

It is likely that the increase of sucrose content in sh2 endosperm activates several signaling pathways, including ABA pathways. Sucrose has been shown to impact ABA signaling, and together they have a demonstrated synergistic impact on gene expression (Rook et al., 2001; Akihiro et al., 2006). Expression profiles change for many transcription factors in *sh2* endosperm, most notably those containing AP2/ERF domains (Figure 4E). Transcription factors with this domain, are responsive to sugars, ABA, and/or ethylene, and frequently have roles integrating these sensing pathways (Weigel, 1995; Finkelstein et al., 1998; Fujimoto et al., 2000). Most are upregulated in *sh2* endosperm (Figure 4E). Some are downregulated, such as an *EREB156* that otherwise enhances starch production in response to sucrose and ABA (Huang et al., 2016). Other sugar modulated AP2/ERF genes upregulated here that would favor sink strength are *ZmWri1* and *ZmWri2*, orthologs of *Wrinkled1* from *Arabidopsis thaliana* (Figure 4E). This AP2/ERF transcription factor is a known regulator of both glycolysis and fatty acid biosynthesis (Baud et al., 2009). In addition, overexpression of the maize *Wri1* increases the oil content of kernels (Shen et al., 2010). The upregulation of *ZmWri1* and *ZmWri2* in *sh2* endosperm correlates with enhanced expression of glycolytic genes, genes for fatty acid biosynthesis, metabolism, and elongation (Table 1, Figure 4D, and Supplementary Table S2), as well as the increased fatty acid levels of *sh2* kernels (Ratcliff et al., 1993).

### A Potential Role for Hexokinase1 in Grain Filling and Composition

Several lines of evidence indicate prominent input from the hexokinase-sensing system in the *sh2* endosperm. Among these is the abundance of hexokinase-responsive genes in the transcriptome (Figures 4D, 5C and Supplementary Tables S5, S8). Most follow a classical feast or famine framework (Koch, 1996) that adjusts source-sink balance, with sugar abundance enhancing mRNAs for sink strength and repressing those for source activity (Supplementary Tables S2, S5 and Figure 4C; Bihmidine et al., 2013; Guan and Koch, 2015). Coexpression network analysis reveals multiple roles for HXK1 in the maize endosperm. Structure of this module links the breadth of previously known HXK1-responsive genes to those for zein storage proteins and to transcription factors that contribute to grain filling (Figure 5C; Aguilera-Alvarado and Sánchez-Nieto, 2017). In the present work, the *HXK1*-coexpression module also correlates most strongly with the sugar and starch content of developing kernels, further emphasizing its importance to grain filling (Figure 5A). As noted above, HXK1 modulates diverse genes (Reviewed in Aguilera-Alvarado and Sánchez-Nieto, 2017), with those upregulated by sugar abundance typically enhancing sink strength relative to source supplies (Bihmidine et al., 2013; Guan and Koch, 2015). In contrast, opposite results, as observed here for N-responses, are expected if a sugar-rich system senses disproportionally limited N-resources (Koch, 1997). Genes for acquisition of N-assimilates are upregulated (those for amino-acid biosynthesis and the GABA shunt), while those for storage in N-sinks are downregulated (zein storage proteins). The decreased expression of genes for zein storage proteins seen here (Supplementary Table S5) is corroborated by other studies of the *sh2* mutant (Sandrine et al., 2020) and of the high-sugar metabolic environments of low-AGPase kernels (Zhang Z. et al., 2019). Work here reveals that *ZmHXK1* is centrally relevant to control of the C:N balance prominent in response to the *sh2* mutation. A still stronger integration of *ZmHXK1* into the control network for C:N balance is evident in the presence of two NAC transcription factors (*ZmNAC12* and *ZmNAC130*) in the same module (Figure 5C and Supplementary Table S8). Among these, previous work by Zhang Z. et al. (2019) indicated that *ZmNAC130* is a key regulator of grain filling and could bind the promoter motif of 50 kD gamma zeins (one of these being a hub gene in the coexpression network identified here) (Figure 5C and Supplementary Table S8). Furthermore, when *ZmNAC130* was downregulated, so was *ZmHXK1.* Taken together, these results indicate that dual action of *ZmHXK1* as a catalytic enzyme and transcription factor upregulates sink metabolism and coordinates zein expression, C:N balance, and the impacts of other transcription factors like *ZmNAC130.*

### Sugar Signaling Plays a Role in the Molecular Response to the *sh2* Mutation

Data here indicate that endosperm perturbation with a *sh2* mutation affects both the sucrose- and hexose-based sensing systems. Differential impacts of sucrose and hexose “states” (Wobus and Weber, 1999) are now recognized as effects of hexokinase (HXK) and trehalose-based (TPS-TPP) signaling systems (Bihmidine et al., 2013; Guan and Koch, 2015). Hexose abundance favors cell division and expansion whereas sucrose enhances differentiation and maturation (Guan and Koch, 2015). In *sh2* endosperm, many hexose sensing pathways including glycolysis and the pentose-phosphate pathway are differentially regulated (Table 1 and Supplementary Table S2). Overall, we see an increase in expression of the enzymes involved in glycolysis and the pentose phosphate pathway in *sh2* endosperm (Table 1, Figure 4D, and Supplementary Table S2). This is likely due to the dramatic increase of sugars and/or changes in flux through these sensing pathways in the kernel (Guan and Koch, 2015). Flux through these pathways is important to many secondary processes as well as regulatory mechanisms. Glycolysis in particular is centrally important in the low-oxygen endosperm of maize (Rolletschek et al., 2005), as well as for sugar signals generated in the first step of this path by hexokinase (Bihmidine et al., 2013). The hexokinase responsiveness of glycolytic genes seen here and elsewhere provides a feed-forward mechanism for enhancing capacity of both glycolytic flux and sensing by hexokinase (Guan and Koch, 2015). In addition to glycolysis, other genes related to oxidative stress and excess respiration are upregulated in *sh2* endosperm (Supplementary Table S5). Upregulation of these pathways is typical of sweet corn backgrounds (de Sousa et al., 2008) as a response to the high-sugar, low oxygen environment of the maize endosperm (Sanclemente et al., 2021). In non-photosynthetic plastids, the main function of glycolysis is to generate carbon skeletons and ATP for processes including fatty acid synthesis (reviewed in Plaxton, 1996). The reducing power for fatty acid synthesis could also be provided by the oxidative pentose phosphate pathway (Kang and Rawsthorne, 1996). Increased flux of sucrose through these pathways probably contributes to the four-fold increase of fatty acids (by weight) in *sh2* endosperm (Ratcliff et al., 1993).

Input into the TPS-TPP system may differ here despite the elevated levels of sucrose in *sh2* mutants. Genes involved in this sensing path and those responsive to its signals are clearly affected (Figures 3, 4B and Supplementary Table S5). In the TPS-TPP sensing system, trehalose is synthesized from UDP-glucose and glucose-6-phosphate by trehalose-6-phosphate synthase (TPS) generating the intermediary, trehalose-6-phosphate (T6P) which is subsequently dephosphorylated by trehalose-6-phosphate phosphatase (TPP) (Henry et al., 2014). This pathway, specifically T6P levels, are implicated in many signaling and regulatory roles and generally indicative of sucrose abundance (Yadav et al., 2014; Oszvald et al., 2018). Here, a kernel-expressed TPP (*ZmTRPP2*) is upregulated in *sh2* endosperm and a class-II TPS (*ZmTRPS11*) is downregulated (Figure 4B). Class-II T6P synthases are non-catalytic proteins that have been conserved throughout evolution of land plants although their function has not yet been identified. Recent studies have implicated their role in transcriptional regulation (Vandesteene et al., 2010; Barraza et al., 2016). Interestingly, one of the hub genes identified here via WGCNA analysis was also a class-II TPS (*ZmTRPS10*) (Supplementary Table S8, black module). This module is highly correlated with overall starch and sugar levels and is enriched for genes with roles in carbohydrate metabolism (Figure 5A and Supplementary Table S7, black module). In addition, Hu S. et al. (2021) identified *ZmTRPS10* (Zm00001eb192680; called *ZmTPS9* in their work) as a QTL for overall starch content and, using mutant analysis, validated this role plus a contribution to seed yield. Taken together, these results indicate that Class-II TPS’s, and specifically *ZmTRPS10*, are central players in the transcriptomic response to the *sh2* perturbation of maize endosperm.

### Impacts of *Sugary Enhancer1* Implicate Prominent Roles for Gibberellin Signaling

Coexpression network analysis identified *Sugary Enhancer1* (*SE1*) as a key regulator of the maize endosperm transcriptome. The *SE1* gene is a long-known effector of starch and sugar metabolism in maize kernels (Gonzales et al., 1976; La Bonte and Juvik, 1990) and is revealed here to be a central regulator of a coexpression module (Figure 5C). This module also includes key regulatory genes such as the GRAS transcription factor, *Scarecrow-like1* (*SCL1*). Also present are multiple genes related to protein-level regulation including a 26S proteasome, a ubiquitin conjugating enzyme, and an F-box protein *ARABIDILLO*. Notably, the 26S proteasome-ubiquitin pathway has a role in gibberellin signaling (Wang and Deng, 2011). Also, the F-box protein, *ARABIDILLO1*, has been shown to repress effects of GA_3_ in Arabidopsis roots (Mu et al., 2010) and aid regulation of seed germination (Moody et al., 2016). To further explore the relationship between *SE1* and gibberellins, we analyzed DEGs in RNA-seq data from *se1* kernels (Zhang X. et al., 2019). In *se1* kernels, *SCL1* and other GA related genes are differentially expressed. In contrast to *sh2* kernels where *SCL1* is downregulated, *SCL1* is upregulated in *se1* kernels. The downregulation of *SCL1* in *sh2* kernels compared to Wt could result from increased ABA response to excess sucrose, as ABA and GA are known to counter one another. Multiple genes for GA-metabolism are upregulated in *se1*, as are GA-activated genes for starch degradation. Taken together, our results indicate that *SE1* could impact starch metabolism via regulation of GA, potentially through control of *SCL1*. The suggested mechanism is further supported by a spectrum of typical GA responses observed here in *se1* kernels, including the increased sugars (specifically maltose), decreased starch (Zhang X. et al., 2019), and upregulation of germination genes such as endoglucanases (MacLeod and Millar, 1962) and β-galactosidases (Giannakouros et al., 1991; Chantarangsee et al., 2007).

## Conclusion

In this study, we utilized near isogenic lines (NILs) of starch biosynthetic mutants *su1* and *sh2* to perturb the maize endosperm transcriptome during progressive developmental stages. Mutation of the *Su1* gene led to minimal changes in the endosperm transcriptome, most of which were took place 28 DAP. In the *sh2* endosperm, we see changes in the regulation of starch and sugar metabolism. Additionally, we identify *ZmHXK1* as a key controller of a coexpression network containing transcription factors (namely *ZmNAC130*) and genes related to grain filling and C:N balance in the endosperm, such as zein-encoding genes. Through a comparison of genetic backgrounds between NILs we also identified putative modifiers of the *sh2* response, including maize *BHLH157* and *MKKK55*, that can potentially be used in sweet corn breeding or for targeted starch production. Coexpression analysis has also provided insight into the role of Class II TPS’s and the Zm*SE1* gene in the maize endosperm. We propose that the previously identified starch content QTL *TRPS10* (Hu S. et al., 2021) functions as a key transcriptional regulator of starch production. We also propose that *ZmSE1* impacts starch metabolism through its function as a key regulator of altering GA signaling in the kernels. It is possible that *se1* kernels begin aspects of germination prematurely through altering GA signaling and derepression of *ZmSCL1*. These results will guide future research to determine the usefulness of the putative starch pathway modifiers and validate the proposed *TRPS10* and *SE1* functions.

## Data Availability Statement

The datasets generated for this study can be found in the NCBI Sequence Read Archive under BioProject PRJNA790930.

## Author Contributions

MR and CF designed the experiments and wrote the manuscript. CF isolated and prepared RNA for sequencing, performed transcriptomic analyses, and performed statistical analysis of kernel carbohydrate data. SB extracted and quantified kernel carbohydrates. WT, KK, LH, KL, and GM contributed to interpretation, discussion, and manuscript review. All authors contributed to the article and approved the submitted version.

## Conflict of Interest

The authors declare that the research was conducted in the absence of any commercial or financial relationships that could be construed as a potential conflict of interest.

## Publisher’s Note

All claims expressed in this article are solely those of the authors and do not necessarily represent those of their affiliated organizations, or those of the publisher, the editors and the reviewers. Any product that may be evaluated in this article, or claim that may be made by its manufacturer, is not guaranteed or endorsed by the publisher.
