# Supplementary figures and images for: Genetic Perturbation of the Starch Biosynthesis in Maize Endosperm Reveals Sugar-Responsive Gene Networks
Source: Front Plant Sci. 2022 Feb 8;12:800326. doi: 10.3389/fpls.2021.800326 (PMC8861272; doi:10.3389/fpls.2021.800326)

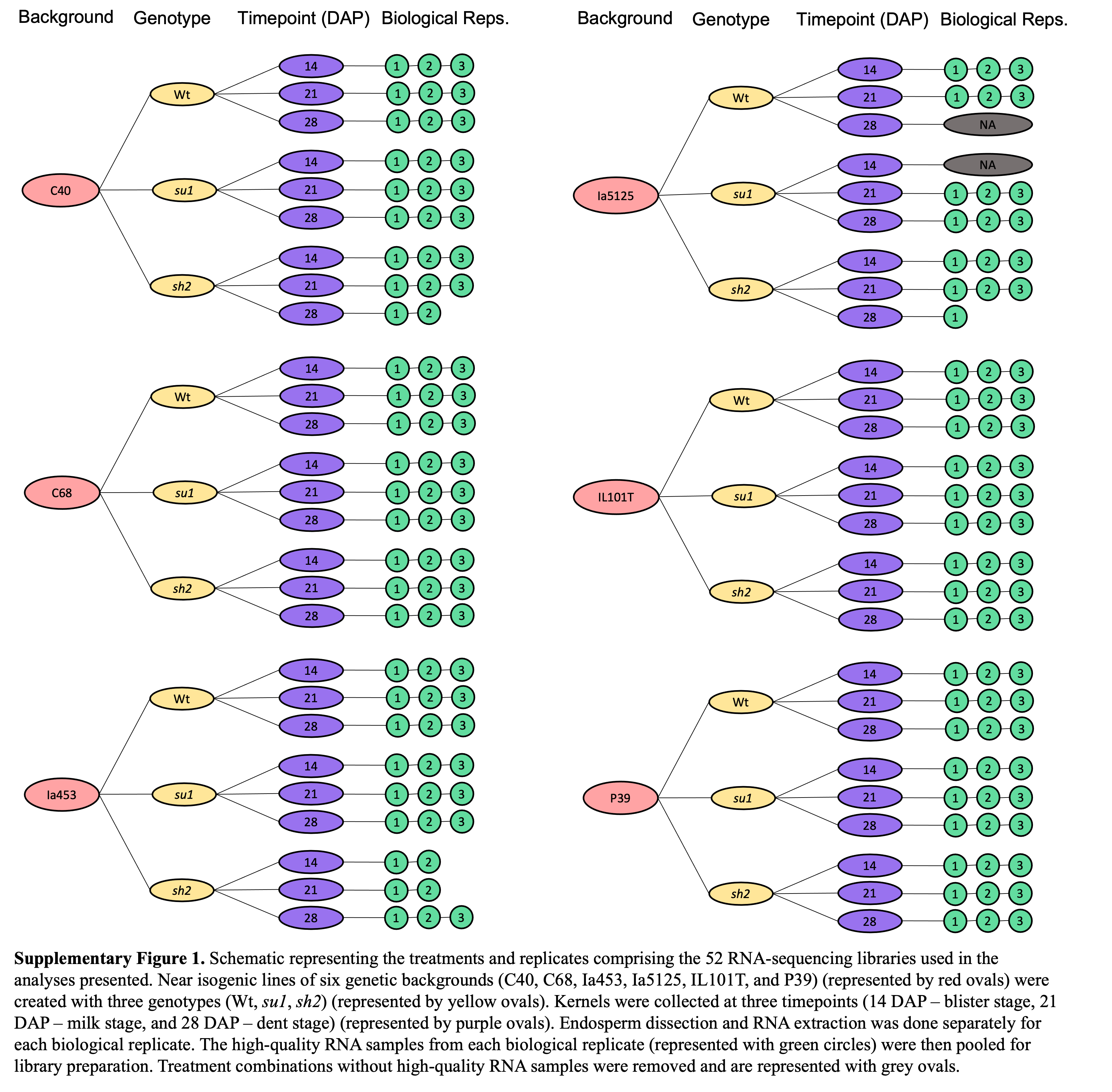

Supplement: Supplementary file 1 [file Data_Sheet_1.zip › 12-27-2021_10.3389-fpls.2021.800326/Image 1_v1.JPEG]

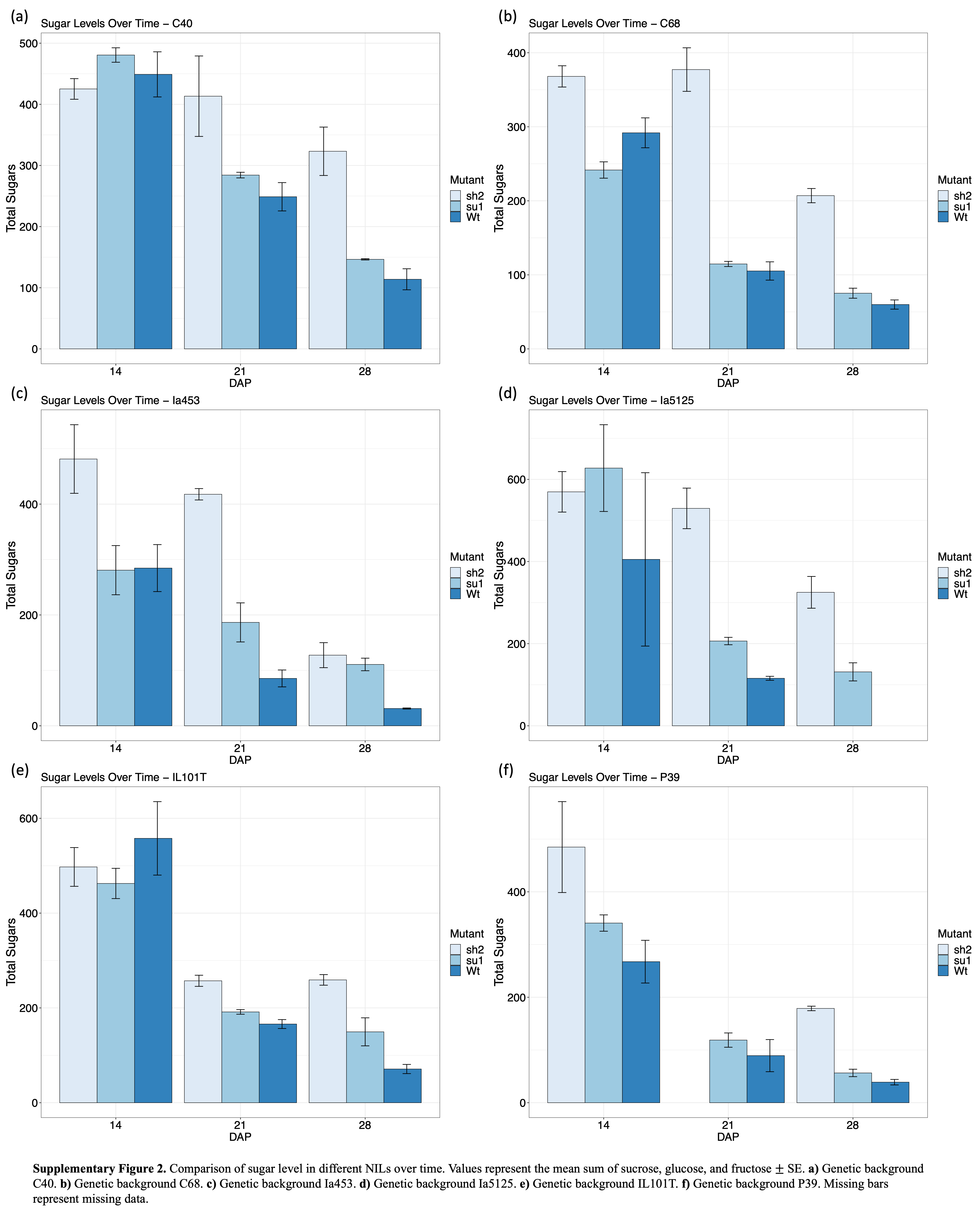

Supplement: Supplementary file 1 [file Data_Sheet_1.zip › 12-27-2021_10.3389-fpls.2021.800326/Image 2_v1.JPEG]
